# CD5L, Macrophage Apoptosis Inhibitor, Was Identified in Epicardial Fat-Secretome and Regulated by Isoproterenol From Patients With Heart Failure

**DOI:** 10.3389/fphys.2020.00620

**Published:** 2020-06-30

**Authors:** Rosa M. Agra-Bermejo, Carla Cacho-Antonio, Adriana Rozados-Luis, Marinela Couselo-Seijas, Angel L. Fernandez, J. M. Martinez-Cereijo, S. B. Bravo, Jose R. Gonzalez-Juanatey, Sonia Eiras

**Affiliations:** ^1^Cardiology Group, Health Research Institute of Santiago de Compostela, Santiago de Compostela, Spain; ^2^Cardiovascular Area and Coronary Unit, University Clinical Hospital of Santiago de Compostela, Santiago de Compostela, Spain; ^3^CIBERCV, Madrid, Spain; ^4^Translational Cardiology Group, Health Research Institute of Santiago de Compostela, Santiago de Compostela, Spain; ^5^Heart Surgery Department, University Clinical Hospital of Santiago de Compostela, Santiago de Compostela, Spain; ^6^Proteomic Unit, Health Research Institute of Santiago de Compostela, Santiago de Compostela, Spain

**Keywords:** epicardial fat, inflammation, heart failure, atrial fibrillation, proteomics

## Abstract

**Objectives:**

Neurohormonal dysfunction, which can regulate epicardial fat activity, is one of the main promoters of atrial fibrillation (AF) in patients with heart failure (HF). Our aim was to study the epicardial fat mediators for AF in patients with HF and its catecholaminergic regulation.

**Methods:**

We have included 29 patients with HF who underwent cardiac surgery and were followed up for 5 years. Released proteins by epicardial adipose tissue (EAT) after isoproterenol treatment were identified by nano-high-performance liquid chromatography (HPLC) and triple time-of-flight (TOF) analysis. Common and differential identified proteins in groups of patients with AF before and after surgery were determined by the FunRich tool. Plasma and epicardial fat biopsy proteins were quantified by western blot.

**Results:**

Our results identified 17 common released proteins by EAT, after isoproterenol treatment, from HF patients who suffered AF or developed new-onset AF during follow-up. Mostly, they were involved on inflammatory response and extracellular matrix. One of them was CD5L, a macrophage apoptosis inhibitor. Its secretion by isoproterenol treatment was validated on western blot. The CD5L levels on epicardial fat were also higher in the group of male patients who present or develop AF (0.44 ± 0.05 vs. 0.18 ± 0.15; *p* < 0.016). However, there were no differences regarding plasma levels.

**Conclusion:**

Our results suggest the role of epicardial fat CD5L as a mediator of AF and its possible paracrine effect by catecholaminergic activity.

## Introduction

The prevalence rates of atrial fibrillation (AF) and heart failure (HF) are growing in parallel because they share the same associated risk factors (obesity, Kenchaiah et al., 2002; Filardo et al., 2009, and aging, Fernandes-Silva et al., 2018). Nowadays, epicardial and pericardial adipose tissues play a central role in the development of both disorders (Thanassoulis et al., 2010; Shin et al., 2011). Epicardial adipose tissue (EAT) is localized around the heart and exerts a direct effect over the myocardium (Venteclef et al., 2015) and coronary arteries (Furuhashi et al., 2016). In addition, in patients with HF, this fat tissue encloses β-adrenergic receptors (Fandiño-Vaquero et al., 2014) and exhibits higher catecholaminergic activity than subcutaneous adipose tissue (SAT) (Parisi et al., 2016). Adrenergic activity promotes contractility and cardiotoxicity through reactive oxygen species production, which are involved in HF progression (Triposkiadis et al., 2009). Thus, the appearance of AF is common in these patients (Workman, 2010) and a consequent cause of HF progression. In adipose tissue, catecholamines upregulate lipolysis (Davies et al., 1982) and protein secretion (Fandiño-Vaquero et al., 2014). Some of the secreted proteins by epicardial fat are involved in fibrosis (Venteclef et al., 2015), which is considered an arrhythmogenic substrate (Platonov, 2017). Since high levels of preoperative catecholamines are associated with postoperative AF (Anderson et al., 2017), we wanted to identify epicardial fat predictors and mediators of AF, regulated by catecholamines, in patients with HF.

## Materials and Methods

### Study Population

We included patients from the following study (Fandiño-Vaquero et al., 2014) with HF diagnosis according to the recommendations of the European Society of Cardiology (Kirchhof et al., 2016; Ponikowski et al., 2016).

At admission, we divided the population into two groups according to the presence of previous AF. They were followed up for 5 years after open-heart surgery. New-onset paroxysmal or persistent AF during follow-up, excluding AF during the first 72 h after surgery, was recorded in a database.

### Samples

The protocol was approved by the Galician Clinical Research Ethics Committee and carried out in accordance with the Declaration of Helsinki. Epicardial fat biopsies (0.1–0.3 g) were obtained before extracorporeal pulmonary circulation from 29 patients who signed informed consent and underwent open-heart surgery (valve replacement and/or coronary artery bypass graft). Blood samples from 12 patients were obtained in EDTA tubes before surgery. The exclusion criteria were previous heart surgery or severe infectious diseases. Instantaneously, the collected samples were placed in a sterile tube with phosphate-buffered saline solution that contained the following (in mM): 0.5 EDTA, 5 KCl, 10 HEPES, 2 MgCl_2_, 10 NaHCO_3_, 0.5 KH_2_PO_4_, 0.5 NaH_2_PO_4_, 10 glucose, 110 NaCl, and 0.16 CaCl_2_ (pH 7.4) (Sigma-Aldrich, San Luis, MO, United States). After washing samples three times, fat pads were split in 100 mg weight and cultured in 24-well plates with 0.5 ml of M199 medium (Lonza Biologics, Porriño, Spain). After 24 h, the medium was replaced, and fat pads were treated or not with isoproterenol at 1 μM for 6 h as previously described. Finally, the supernatants and fat tissues were collected and stored at −80°C until use.

### Protein Identification

Secretomes from isoproterenol-treated fat pads of 11 patients were divided and filtrated by two different Amicon Ultra 0.5-ml columns (>50 and 10–50 kDa) (Merck Millipore, Billerica, MA, United States). One fraction (named A) contained proteins heavier than 50 kDa, and the other fraction (named B) had proteins with a molecular weight between 10 and 50 kDa. Then, proteins were loaded and separated by sodium dodecyl sulfate polyacrylamide gel electrophoresis (SDS-PAGE). After gels were stained with SYPRO Ruby (Bio-Rad), protein bands were digested with trypsin (Shevchenko et al., 1996), and peptides were separated by reverse-phase chromatography. Gradient was achieved using a micro liquid chromatography system (Eksigent Technologies nanoLC 400, SCIEX) coupled to high-speed triple time-of-flight (TOF) 6600 mass spectrometer (SCIEX) with a micro flow source. The silica-based reverse-phase column was YMC-TRIART C18 150 × 0.30 mm with a 3-mm particle size and 120-Å pore size (YMC Technologies, Teknokroma), and the trap column was YMC-TRIART C18 (YMC Technologies, Teknokroma) with a 3-mm particle size and 120-Å pore size, switched online with the analytical column. A solution of 0.1% formic acid in water at 10 μl/min was loaded in the pump, and the micro-pump provided a flow rate of 5 μl/min, and it was operated under gradient elution (0.1% formic acid in water was mobile phase A and 0.1% formic acid in acetonitrile was mobile phase B). Peptides were separated using a 90-min gradient ranging from 2 to 90% mobile phase B (mobile phase A: 2% acetonitrile, 0.1% formic acid; mobile phase B: 100% acetonitrile, 0.1% formic acid). The injection volume was 4 μl, and data were acquired in a triple TOF 6600 system (SCIEX, Foster City, CA, United States) using a data-dependent workflow. Source and interface conditions were as follows: ion spray voltage floating (ISVF) 5,500 V, curtain gas (CUR) 25, collision energy (CE) 10, and ion source gas 1 (GS1) 25. The instrument was operated with Analyst TF 1.7.1 software (SCIEX, United States). Switching criteria were set to ions greater than a mass-to-charge ratio (*m*/*z*) of 350 and smaller than *m*/*z* 1,400 with a charge state of 2–5, mass tolerance of 250 ppm, and an abundance threshold of more than 200 counts (cps). Former target ions were excluded for 15 s. The instrument was automatically calibrated every 4 h using tryptic peptides from beta galactosidase as an external calibrant.

**FIGURE 1:**
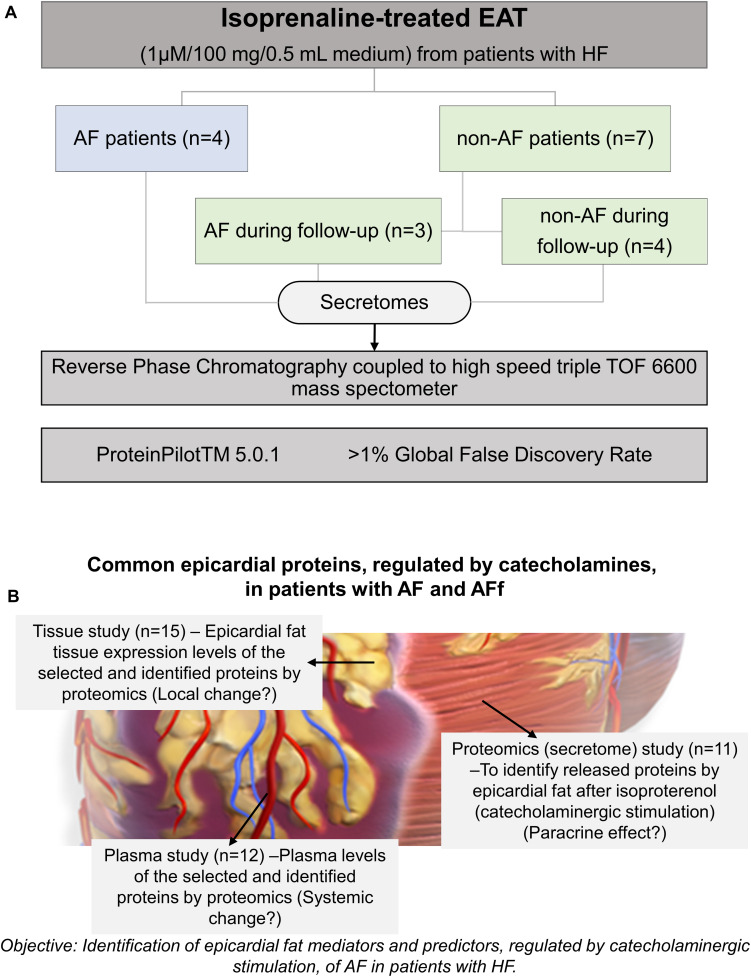
Study design. Epicardial adipose tissue (EAT) samples were obtained from 11 heart failure (HF) patients undergoing open-heart surgery. Samples were submitted to isoprenaline treatment. Secretome protein analysis was performed after a 5-year follow-up. Samples were classified according to atrial fibrillation absence (non-AF), AF onset, or previous AF. **(A)** Design for validating the identified proteins and their changes on tissue (local effect), secretome (paracrine effect) or plasma (systemic effect) **(B)**.

After MS/MS analysis, data files were processed using Protein Pilot TM 5.0.1 software from SCIEX which uses the algorithm Paragon^TM^ for database search and Progroup^TM^ for data grouping. Data were searched using a human-specific UniProt database. Then, identified proteins were grouped with the functional enrichment analysis tool (FunRich) in the presence/absence of AF or presence/absence of AF during follow-up. The design of the study is shown in Figure 1A. Then, the selected proteins, after being identified, were tested on EAT and plasma to study the local or systemic changes (Figure 1B).

### Tissue Homogenization and Protein Analysis

Stored fat tissues (100 mg) from 15 patients were lysed with 0.3 ml of RIPA buffer [25 mM Tris–HCl, pH = 7.6, 150 mM NaCl, 1% (C_2_H_4_O) nC_14_H_22_O, 1% C_24_H_39_NaO_4_, and 0.1% NaC_12_H_25_SO_4_]. All of them were from Sigma-Aldrich. Protease and phosphatase inhibitors (complete tables and PhosStOP easy pack from Roche Diagnostics, Basilea, CH) were added to RIPA buffer. Then, tissues were homogenized in a tissue lyser with steel beads (Qiagen, Hilden, DE) for 3 min at 30 Hz. Lysed tissues were centrifuged at 12,000 × *g* for 10 min, and the lipid layer was discarded. Proteins were purified with a 2-D Clean-Up Kit (GE Healthcare, Chicago, IL, United States) following the manufacturer’s recommendations. Finally, 20 mg per tissue was separated with 8% SDS-PAGE and transferred to fluorescence polyvinylidene difluoride (PVDF) membranes (Merck Millipore, Burlington, MA, United States), at 300 mA for 90 min. The primary antibody against CD5L (1:1,000) was incubated in agitation overnight at 4°C (Santa Cruz Biotechnology, Dallas, TX, United States) after being blocked with 3% bovine serum albumin in Tris-buffered saline solution with Tween 20 (TBST), containing 20 mM Tris–HCl, pH = 7.6; 150 mM NaCl; and 0.% Tween 20. To remove the excess primary antibody, membranes were washed three times for 10 min with TBST. Finally, protein was detected in a fluorescence scanner (Typhoon FLA 9500, GE Healthcare) after membrane incubation with fluorescent anti-mouse secondary antibody Alexa 532 (Thermo Fisher). Densitometry of CD5L and IgG bands were determined by Quantity One software (Bio-Rad, Hercules, CA, United States). CD5L values were represented as the CD5L/IgG ratio in arbitrary units (a.u.). Plasma from 12 patients was obtained after blood centrifuging at 1,800 × *g*. Then, IgG and albumin were depleted with a mixed bed of Cibacron blue/protein A gel following the manufacturer’s protocol (Thermo Scientific). At the end of the protocol, plasma was diluted seven times. After plasma filtration through 3-kDa columns (Sigma-Aldrich), it was diluted three times, and 10 μl was separated by SDS-PAGE and transferred to PVDF membranes that were incubated with CD5L antibody as described above. CD5L levels were referenced to values of the positive control (same sample on every western blot).

CD5L was also determined in secretomes and biopsies from three independent patients with or without isoproterenol treatment following the same previously described protocol.

### Statistical Analysis

After protein identification, false discovery rate was performed using a nonlineal fitting method displaying only those results that reported ≥1% global false discovery rate (Shilov et al., 2007; Tang et al., 2008). Clinical continuous variables were presented as mean ± standard deviation, and categorical variables were presented as frequency and percentage. Comparison between groups was performed with unpaired *t*-test or Pearson’s χ^2^ test. Differences regarding two groups were performed by unpaired *t*-test analysis. All analyses were performed using SPSS 19.0. (Software SPSS Inc., Chicago, IL, United States).

## Results

### EAT-Released Proteins, After β-Adrenergic Stimulus, From HF Patients With and Without Previous AF Who Did or Did Not Develop AF

Proteomics studies were performed with samples from 11 patients (seven without AF and four with AF that developed during follow-up). The main clinical characteristics are detailed in Table 1A.

**TABLE 1A:** Clinical characteristics of proteomic study with respect to AF.

	**With AF (*n* = 4)**	**Without AF (*n* = 7)**
Age years (SD)	72 (3)	68 (12); *p* = 0.501
BMI, kg/m^2^ (SD)	27 (5)	31 (4); *p* = 0.126
Heart rate (bpm)	65 (10)	76 (12); *p* = 0.186
Gender (male/female)	1/3	2/5; *p* = 0.898
CAD (no/yes)	3/1	3/4; *p* = 0.303
NYHA (I/II/III-IV)	0/2/2	2/4/1; *p* = 0.308
T2DM (no/yes)	3/1	6/1; *p* = 0.658
HTA (no/yes)	2/2	2/5; *p* = 0.477
DLP (no/yes)	3/1	1/6; *p* = 0.044*
Aortic valve replacement (no/yes)	3/1	2/5; *p* = 0137
Mitral valve replacement (no/yes)	2/2	6/1; *p* = 0.201
LVEF (%)	49 (15)	61 (15); *p* = 0.223
Diuretics (no/yes)	3/1	2/4; *p* = 0.197
β-Blockers (no/yes)	2/2	3/3; *p* = 1.000
Statins (no/yes)	2/2	2/4; *p* = 0.598
ACEi (no/yes)	2/2	4/2; *p* = 0.598
ARB (no/yes)	2/2	3/3; p = 1.000
Antiarrhythmic class III (no/yes)	2/2	6/0; *p* = 0.053

	**With AFf (*n* = 3)**	**Without AFf (*n* = 4)**

Age years (SD)	68 (6)	68 (16); *p* = 0.975
BMI, kg/m^2^ (SD)	32 (4)	31 (4); *p* = 0.699
Heart rate (bpm)	76 (5)	75 (17); *p* = 0.976
Gender (male/female)	0/3	2/2; *p* = 0.147
CAD (no/yes)	2/1	1/3; *p* = 0.270
NYHA (I/II/III)	0/3/0	2/1/1; *p* = 0.140
T2DM (no/yes)	2/1	4/0; *p* = 0.212
HTA (no/yes)	1/2	1/3; *p* = 0.809
DLP (no/yes)	0/3	1/3; p = 0.350
Aortic valve replacement (no/yes)	1/2	1/3; *p* = 0.809
Mitral valve replacement (no/yes)	2/1	4/0; *p* = 212
LVEF (%)	65 (6)	58 (29); *p* = 0.567
Diuretics (no/yes)	0/2	2/2; *p* = 0.221
β-Blockers (no/yes)	1/1	2/2; *p* = 1.000
Statins (no/yes)	1/1	1/3; *p* = 540
ACEi (no/yes)	2/0	2/2; *p* = 221
ARB (no/yes)	1/1	2/2; *p* = 1.000

*BMI, body mass index; CAD, coronary artery disease; NYHA, New York Heart Association; T2DM, type 2 diabetes mellitus; HTA, hypertension arterial; DLP, dyslipidemia; LVEF, left ventricle ejection fraction; ACEi, angiotensin-converting enzyme inhibitors; ARB, angiotensin receptor blockers; AFf, AF during follow-up; AF, atrial fibrillation. ^∗^*p* < 0.05 statistical significance.*

**FIGURE 2:**
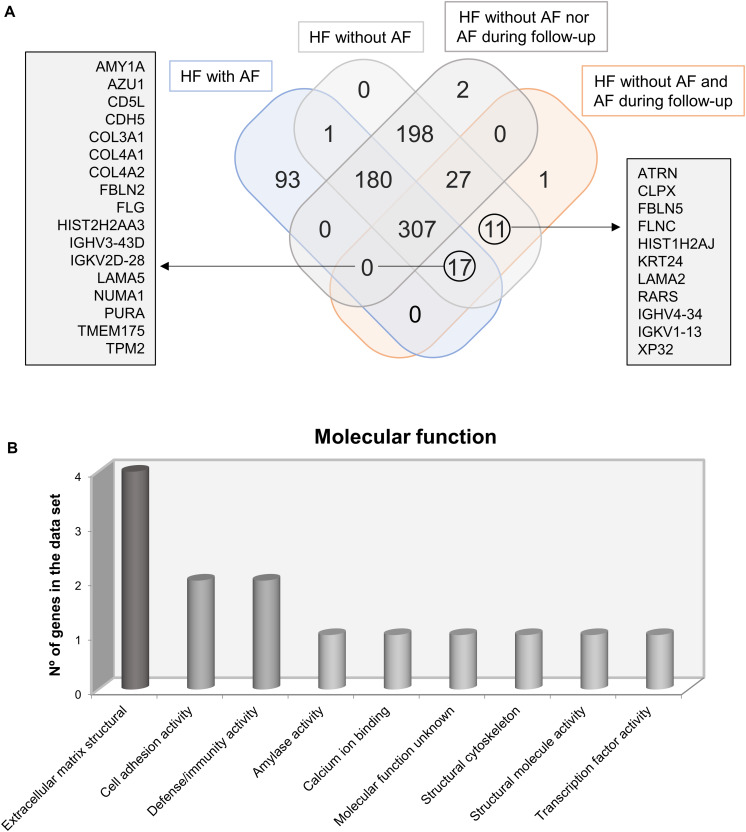
Venn diagram determines common and differential identified proteins in epicardial adipose tissue (EAT) secretomes from patients who suffer or not from atrial fibrillation (AF) or will develop or not AF after follow-up. Common proteins of EAT secretome from patients with AF and those who develop AF are named in the right square **(A)**. Graph represents molecular function of identified proteins on EAT secretomes from AF patients and those who develop AF **(B)**.

After identifying proteins on each EAT secretome fraction, we found a mean of 328 ± 150 proteins with a molecular weight higher than 50 kDa (fraction A) and a mean of 55 ± 10 proteins with a molecular weight between 10 and 50 kDa (fraction B). Identified proteins on A and B per patient were analyzed by the FunRich tool. The Venn diagram determined 334 common proteins between EAT secretomes from patients with HF who did or did not develop new-onset AF during follow-up. However, only 29 EAT-secreted proteins were differentially identified between them (Figure 2).

The comparison between patients with or without AF identified 488 common proteins and 93 proteins specifically in EAT secretomes from AF patients. After testing the common proteins between patients with AF or those who develop new-onset AF during follow-up, we found 17 proteins (Figure 2A). Some of the identified proteins are typically expressed in neutrophils [azurocidin (AZU1)], in macrophages and inflamed tissues CD5 molecule like [CD5L], and in blood vessels [cadherin-5 (CDH5) and tropomyosin (TPM2)]. The rest of the proteins were mainly a compound of the extracellular matrix [collagens 3 and 4 (COL3 and COL4, respectively), fibulin-2 (FBLN2), and laminin subunit alpha-5 (LAMA5)]. Thus, after classifying proteins according their molecular functions, we observed that most of them were compounds of the extracellular matrix or cell adhesion junctions. But, also, we have to emphasize the presence of two proteins which are involved in immune defense (AZU1 and CD5L) (Figure 2B). In addition, we have to highlight the presence of attractin (ATRN), which regulates the chemoattractant activity during the inflammatory response, in those patients who develop new-onset AF during follow-up.

### Differential Levels Among EAT Biopsies, EAT Secretomes, and Plasma

**FIGURE 3:**
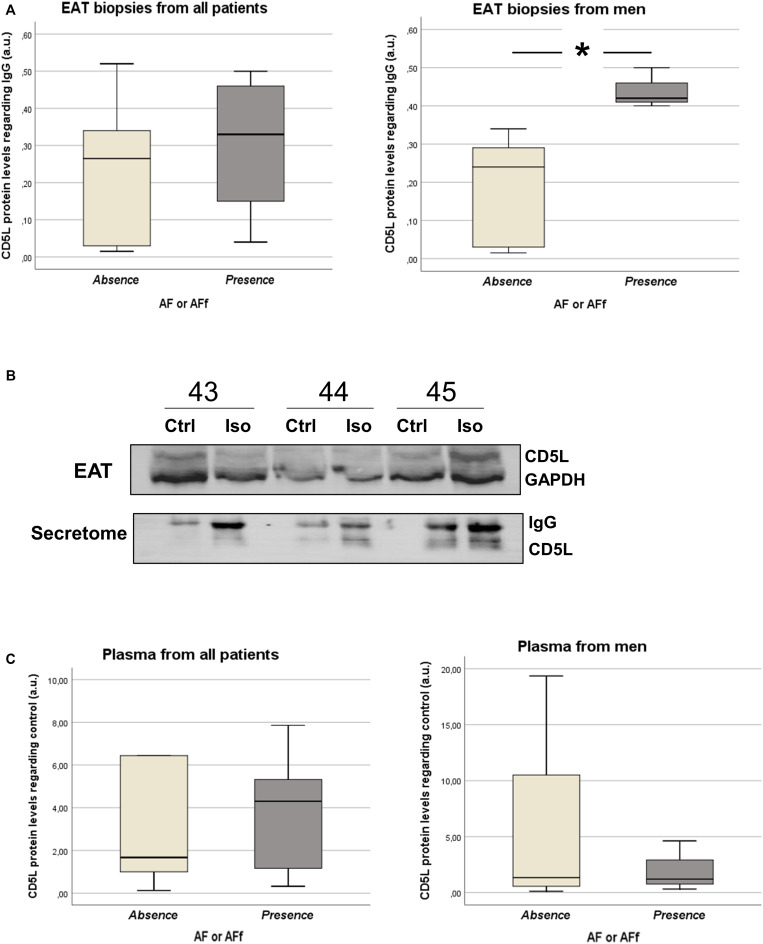
Box plots represent the CD5L levels in epicardial adipose tissue (EAT) biopsies from patients with sinus rhythm (SR) and atrial fibrillation (AF) or new-onset AF after follow-up **(A)**. Western blot for analyzing CD5L levels in EAT biopsies and supernatants from three patients after isoproterenol treatment **(B)**. Western blots for analyzing plasma CD5L levels in patients with SR or AF and new-onset AF after follow-up **(C)**.

After identifying epicardial fat proteins, whose secretion is regulated by isoproterenol treatment, on patients with AF or AF during follow-up, we wanted to study their own expression levels on tissue. Because collagen (COL) (Kallergis et al., 2008) or fibulins (FBLNs) (García et al., 2011) were already described to be associated with AF, we decided to test CD5L in EAT biopsies from 15 patients (clinical characteristics in Table 1B). Our data showed that CD5L levels did not reach the difference with statistical significance between patients with and without AF or who suffered AF during follow-up (0.46 ± 0.56 vs. 0.24 ± 0.19). However, after splitting the population based on sex, we observed a higher levels in those male patients who had or developed AF (0.44 ± 0.05 vs. 0.18 ± 0.15; *p* < 0.016) (Figure 3A). Whereas it is secreted by isoproterenol on proteomics study, we confirmed by western blot the same result (Figure 3B). We tried to test if CD5L plasma levels were modified with AF presence. We have included samples from 12 patients (Table 1C). However, we did not observe differences with statistical significance between patients with AF or those who develop AF during follow-up and those who are non-AF (Figure 3C).

**TABLE 1B:** Clinical characteristics of tissue CD5L study with respect to A.

	**With AF or AFf (*n* = 9)**	**Without AF (*n* = 6)**
Age years (SD)	68 (10)	66 (16); *p* = 0.788
BMI, kg/m^2^ (SD)	30 (6)	31 (4); *p* = 0.938
Heart rate (bpm)	69 (15)	69 (20); *p* = 0.995
Gender (male/female)	4/5	5/1; *p* = 0.138
CAD (no/yes)	2/7	5/1; *p* = 0.020*
NYHA (I/II/III-IV)	2/7/0	1/3/2; p = 0.176
T2DM (no/yes)	7/2	5/1; *p* = 0.792
HTA (no/yes)	5/4	2/4; *p* = 0.398
DLP (no/yes)	5/3	1/5; *p* = 0.086
Aortic valve replacement (no/yes)	2/7	2/4; *p* = 0.634
Mitral valve replacement (no/yes)	7/2	5/1; *p* = 0.796
LVEF (%)	55 (10)	57 (22); *p* = 0.995
Diuretics (no/yes)	3/5	2/4; p = 0,872
β-Blockers (no/yes)	5/3	1/5; *p* = 0.086
Statins (no/yes)	5/3	2/4; *p* = 0.280
ACEi (no/yes)	5/3	4/2; *p* = 0.872
ARB (no/yes)	7/1	3/3; *p* = 0.124
Antiarrhythmic class III (no/yes)	7/1	6/0; *p* = 0.369

*BMI, body mass index; CAD, coronary artery disease; NYHA, New York Heart Association; T2DM, type 2 diabetes mellitus; HTA, hypertension arterial; DLP, dyslipidemia; LVEF, left ventricle ejection fraction; ACEi, angiotensin-converting enzyme inhibitors; ARB, angiotensin receptor blockers; AFf, AF during follow-up; AF, atrial fibrillation. ^∗^*p* < 0.05 statistical significance.*

**TABLE 1C:** Clinical characteristics of plasma CD5L study with respect to AF.

	**With AF or AFf (*n* = 7)**	**Without AF (*n* = 5)**
Age years (SD)	66 (12)	68 (13); *p* = 0.868
BMI, kg/m^2^ (SD)	33 (8)	28 (5): *p* = 0.304
Heart rate (bpm)	66 (10)	67 (17); *p* = 0.906
Gender (male/female)	4/3	1/4; *p* = 0.198
CAD (no/yes)	4/3	2/3; *p* = 558
NYHA (I/II/III-IV)	0/4/3	0/3/2; *p* = 0.921
T2DM (no/yes)	2/5	4/1; *p* = 0.079
HTA (no/yes)	3/4	2/3; *p* = 0.921
DLP (no/yes)	3/4	3/2; *p* = 0.558
Aortic valve replacement (no/yes)	3/4	1/4; *p* = 0.408
Mitral valve replacement (no/yes)	4/3	3/2; *p* = 0.921
LVEF (%)	56 (8)	66 (20); *p* = 0.333
Diuretics (no/yes)	2/4	0/5; *p* = 0.154
β-Blockers (no/yes)	3/2	3/1; *p* = 0.635
Statins (no/yes)	1/5	3/2; *p* = 0.137
ACEi (no/yes)	4/2	2/3; *p* = 0.376
ARB (no/yes)	4/2	4/1; *p* = 0.621
Antiarrhythmic class III (no/yes)	4/2	5/0; *p* = 0.154

*BMI, body mass index; CAD, coronary artery disease; NYHA, New York Heart Association; T2DM, type 2 diabetes mellitus; HTA, hypertension arterial; DLP, dyslipidemia; LVEF, left ventricle ejection fraction; ACEi, angiotensin-converting enzyme inhibitors; ARB, angiotensin receptor blockers; AFf, AF during follow-up; AF, atrial fibrillation. ^∗^*p* < 0.05 statistical significance.*

## Discussion

For the first time, we identified epicardial fat mediators of AF, whose secretion is regulated by β-adrenergic stimulation, on patients with HF. The combination of HF and AF disease increments the death probability (Middlekauff et al., 1991), in part, due to the fact that the high adrenergic activity contributes to its progression (Workman, 2010). Catecholamines have a binding site on the heart, skeletal muscle, and adipose tissue (Sillence et al., 2005). The last one can be localized over the myocardium and incremented during obesity, being one of the major causes of ventricle dysfunction and atrial dimensions (Iacobellis et al., 2007). Thus, adipokine regulation by adrenergic activity on adipose tissue might be involved in adverse consequences of the myocardium structure (Packer, 2018). In line with this, several of them were found to favor fibrosis (Venteclef et al., 2015; Fukui et al., 2017) and AF. We mainly identified epicardial fat proteins involved in the extracellular matrix, inflammation, and cell adhesion. They were common in supernatants of epicardial fat from patients who developed AF during follow-up with or without AF prior to surgery. These results suggest proteins with a predictor and mediator role of AF which are regulated by catecholaminergic activity on epicardial fat. One of them, CD5L, was upregulated on EAT in patients with AF or those who develop AF. Its secretion by catecholaminergic stimulation might increase its paracrine effect over the myocardium or other adjacent tissues. In human atrial tissues, the higher presence of COL type 3 was associated with higher macrophage migration inhibitory factor (MIF) in patients with AF (Xue et al., 2018). Thus, fibrosis and inflammation lead to structural remodeling. Although we did not identify MIF as a differential factor, we detected CD5L. This protein, named the autophagy inhibitor macrophages, is a soluble receptor of macrophages that can be circulating at high concentrations in the blood with immunoglobulin M (IgM) (Sanjurjo et al., 2015). However, after testing their plasma levels, we did not find differences regarding AF. Thus, a local inflammatory effect on epicardial fat might explain the differences in plasma or tissue CD5L behavior. Even obesity, which is associated with a high macrophage infiltration in adipose tissue (Weisberg et al., 2003), might be a risk factor with an important contribution of CD5L levels on epicardial fat. It can also explain the identification of attractin protein, which is incremented in obesity, as a risk factor of new-onset AF. Thus, higher CD5L levels in plasma might be coming from the adipose tissue secretion induced by high catecholamine activity. We tried to corroborate this hypothesis, and CD5L was analyzed in supernatants (García et al., 2011) and biopsies from epicardial fat after catecholamine treatment. Our results confirmed the secretion of CD5L by EAT after isoproterenol treatment. This molecule might activate the toll-like receptor 4/nuclear factor-kappa B (NF-κB) pathway on the adjacent cells and the production of proinflammatory cytokines. Other identified proteins in EAT secretomes from patients with HF and AF were the COLs. It is known that their plasma levels were found to be biomarkers of AF (Duprez et al., 2018). However, plasma CD5L was not an AF risk factor. Maybe, the combination of all identified proteins (markers of chemotactic activity, macrophage viability, neutrophil activity, adhesion, and extracellular matrix) will be useful for stratifying patients with high catecholaminergic activity in patients with EAT, HF, and AF. Some studies are trying to looking for proteomic chips as new biomarkers of AF risk (Lind et al., 2017) because HF is a syndrome with multiple involved pathways or mechanisms. However, several data regarding proinflammatory monocytes (Eiras et al., 2017) or macrophage activity (CD5L) suggest the important role of inflammatory response of EAT on HF progression.

## Limitations

This is a substudy of the previous one (Fandiño-Vaquero et al., 2014), and the sample size left with quality after 5 years’ follow-up is too low. Quantification of CD5L was tested by a semiquantitative technique, western blot. A low percentage of patients developed new-onset AF during long-term follow-up. Plasma and tissue were obtained from different patients. Catecholamine levels on each patient were not analyzed or registered. AF asymptomatic was not detected or registered.

## Conclusion

Although further studies are needed, our results suggest that CD5L levels in EAT are mediators of AF in male patients with HF. Further studies are needed to understand its paracrine role in the myocardium after catecholaminergic activity.

## Data Availability Statement

The proteomic data has been deposited into Figshare (https://figshare.com/articles/Epicardial_fat-secretome_from_heart_failure/12307433).

## Ethics Statement

The studies involving human participants were reviewed and approved by Galician Clinical Research Ethics Committee. The patients/participants provided their written informed consent to participate in this study.

## Author Contributions

RA-B and CC-A have included and characterized patients. AR-L and MC-S have performed the “*ex vivo*” assays, groups of patients and validation. JM-C and AF have included patients undergoing open heart surgery. SB has performed the mass spectrometry. JG-J and SE have designed the protocols. All the authors have contributed to the writing of the manuscript.

## Conflict of Interest

The authors declare that the research was conducted in the absence of any commercial or financial relationships that could be construed as a potential conflict of interest.
